# Intermixing of MoS_2_ and WS_2_ photocatalysts toward methylene blue photodegradation

**DOI:** 10.3762/bjnano.15.68

**Published:** 2024-07-05

**Authors:** Maryam Al Qaydi, Nitul S Rajput, Michael Lejeune, Abdellatif Bouchalkha, Mimoun El Marssi, Steevy Cordette, Chaouki Kasmi, Mustapha Jouiad

**Affiliations:** 1 Laboratory of Physics of Condensed Mater, University of Picardie Jules Verne, 80039 Amiens, France; 2 Technology Innovation Institute, Abu Dhabi P.O. Box 9639, United Arab Emirates

**Keywords:** methylene blue, MoS_2_/WS_2_ composite, photocatalysis, photodegradation, transition-metal dichalcogenides

## Abstract

Visible-light-driven photocatalysis using layered materials has garnered increasing attention regarding the degradation of organic dyes. Herein, transition-metal dichalcogenides MoS_2_ and WS_2_ prepared by chemical vapor deposition as well as their intermixing are evaluated for photodegradation (PD) of methylene blue under solar simulator irradiation. Our findings revealed that WS_2_ exhibited the highest PD efficiency of 67.6% and achieved an impressive PD rate constant of 6.1 × 10^−3^ min^−1^. Conversely, MoS_2_ displayed a somewhat lower PD performance of 43.5% but demonstrated remarkable stability. The intriguing result of this study relies on the synergetic effect observed when both MoS_2_ and WS_2_ are combined in a ratio of 20% of MoS_2_ and 80% of WS_2_. This precise blend resulted in an optimized PD efficiency and exceptional stability reaching 97% upon several cycles. This finding underscores the advantageous outcomes of intermixing WS_2_ and MoS_2_, shedding light on the development of an efficient and enduring photocatalyst for visible-light-driven photodegradation of methylene blue.

## Introduction

Water contamination has become a pressing global concern, threatening ecosystems, agriculture, and human well-being [1–2]. The massive industrialization has dramatically contributed to water pollution, which has prompted policymakers to put in place corrective actions for the development of efficient strategies for water treatment [3]. Following these measures, various technologies have proven their efficacy for water depollution, including adsorption and photocatalysis, and are often utilized for heavy metals, pharmaceuticals, pesticide removal, or synthetic dye degradation [4–6]. For instance, methylene blue (MB), which is considered one of the most used synthetic organic dyes in various industrial and medical applications, poses serious risks as a pollutant to water resources [7]. Indeed, MB is a potential carcinogen and mutagen, directly threatening human health if present in drinking water or in aquatic organisms [8]. In this context, photocatalysis has emerged as a reliable and environmentally friendly solution for MB photodegradation (PD) as it only consumes renewable energy, prevents the formation of secondary waste, and is a cost-effective technology. By harnessing impinging photons, the photocatalytic degradation of pollutants takes place at the interface between the photocatalyst surface and the MB-contaminated electrolyte. The photon energy is the driving force for breaking down the MB compound leading to its removal [9]. Typically, semiconductor-based photocatalysts, such as TiO_2_, ZnO_2_, and some other high-bandgap transition-metal dichalcogenides (TMD) have shown their ability to efficiently degrade the activated MB by irradiation [10–11].

Recently, TMD such as MoS_2_ and WS_2_, have displayed remarkable potential as cocatalysts. Their catalytic properties can be tailored based on their crystal structure, their surface area, and their morphology [12–13]. When TMD catalysts are intermixed, they form semiconductor–semiconductor junctions, enhancing their photocatalytic properties by promoting charge separation and electron transport [14–15]. At each stage of the photocatalytic process sequence, the intermixing of TMD materials is intended to efficiently enhance light absorption, photogeneration of charge carriers, and activation of the surface redox reaction [16–17]. Furthermore, TMD materials are known to possess favorable electrical conductivity, which allows them to serve as sites for trapping photogenerated charges. This, in turn, facilitates the collection of charge carriers [18] leading to interesting photodegradation properties [19–20]. During the photochemical reaction process, the light excitation induces the generation of electron–hole pairs (EHPs) [21]. The generated EHPs react with oxygen and water molecules to produce highly reactive species, such as hydroxyl radicals, which oxidize and degrade MB contaminants. Hence, evaluating the PD processes in the case of MoS_2_ and WS_2_ as TMD materials is crucial for optimizing their functionalities to design novel materials and devices with improved PD stability and durability [8–10].

Recently, researchers have investigated the use of MoS_2_ as a photocatalyst for the degradation of MB. They demonstrated that the MoS_2_–GO compound exhibited interesting PD performances, with over 99% degradation of MB achieved within 60 min under visible light exposure by using 10 mg of the catalyst to degrade 10 mg/L of MB [22]. Other works have shown that the MoS_2_–ZnO composite achieved 97% of MB photodegradation in ≈30 min under visible irradiation by using 250 mg/L of the catalyst to degrade 10 mg/L of MB [23]. Moreover, when MoS_2_ is mixed with SnO_2_, the MB photodegradation reaches up to ≈99.5% within 5 min. This rapid degradation occurred when 400 mg/L of the catalyst was used to degrade 3.2 mg/L of MB [24]. These results concerned materials fabricated using the hydrothermal technique, involving multiple processing stages. This ends up increasing the overall costs of production and creates a real challenge [25]. One can note the very high amount of catalyst used to degrade a small MB concentration. Besides, other studies have shown that WS_2_/polypyrrole composites synthesized by oxidative polymerization achieved a photodegradation efficiency of 96.15% in 180 min by using 100 mg of the catalyst to degrade 5 mg/L of MB [25]. Nevertheless, most of the reported studies concerned the use of complex heterostructure-based devices and a very high quantity of catalysts, which is not convenient for potential commercial upscaling.

In the present work, we report on a systematic study carried out to assess MB photodegradation using chemically vapor-deposited intermixed MoS_2_/WS_2_ at different ratios. The obtained results are compared and discussed based on their respective photodegradation yield, their physical properties, and their evolving microstructures.

## Results and Discussion

### Structural analysis

**Figure 1:**
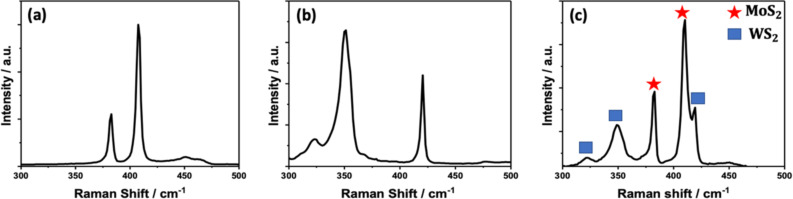
Raman spectroscopy for a) MoS_2_, b) WS_2_, and c) MoS_2_/WS_2_ composite.

Raman spectroscopy analysis of the exfoliated samples revealed prominent vibrational modes of hexagonal 2H-MoS_2_, 2H-WS_2_, and mixture of both phases, represented by E^1^_2g_ at 382 cm^−1^ and A^1^_g_ at 410 cm^−1^, respectively, for MoS_2_, 350 cm^−1^. It can be further resolved into two sub-peaks at 324 cm^−1^ and 351 cm^−1^, corresponding to the 2LA(M) and E^1^_2g_ modes, the A^1^_g_ mode at 420 cm^−1^ for WS_2_, and the presence of combined vibration modes for the composite MoS_2_/WS_2_ as shown in Figure 1.

Interestingly, the positions of the E^1^_2g_ and A^1^_g_ vibrational modes in the composite sample did not exhibit any noticeable shifts compared to the observed peaks in individual samples as reported in previous studies [26].

**Figure 2:**
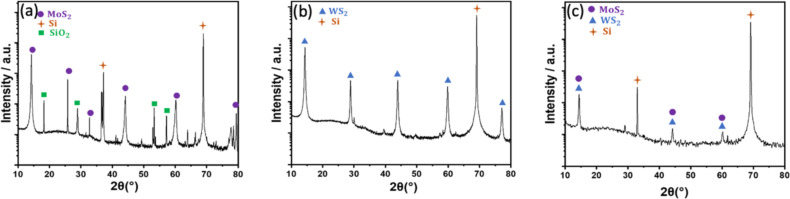
XRD diagrams for a) MoS_2_, b) WS_2_, and c) MoS_2_/WS_2_ composite.

The X-ray diffraction (XRD) diagram shown in Figure 2a exhibits the diffraction peaks at 14.25°, 25.81°, 32.15°, 44.13°, and 60.21° positions, corresponding to (002), (004), (103), (006), and (008) planes of hexagonal 2H-MoS_2_. Likewise, Figure 2b displays the diffraction peaks at 14.3°, 28.8°, 43.9°, 59.8°, and 77.13° positions, attributed to (002), (004), (006), (008), and (0010) planes of 2H-WS_2_, respectively.

The XRD diagram depicted in Figure 2c reveals the combination of peaks arising from both 2H-MoS_2_ and 2H-WS_2_, confirming the successful intermixing of the MoS_2_/WS_2_ composite. The sharp shape of the diffraction peaks suggests a very good crystallinity of the fabricated materials. The recurring additional peaks observed in all XRD diagrams at ≈37° and ≈69° positions are due to the silicon substrate.

**Figure 3:**
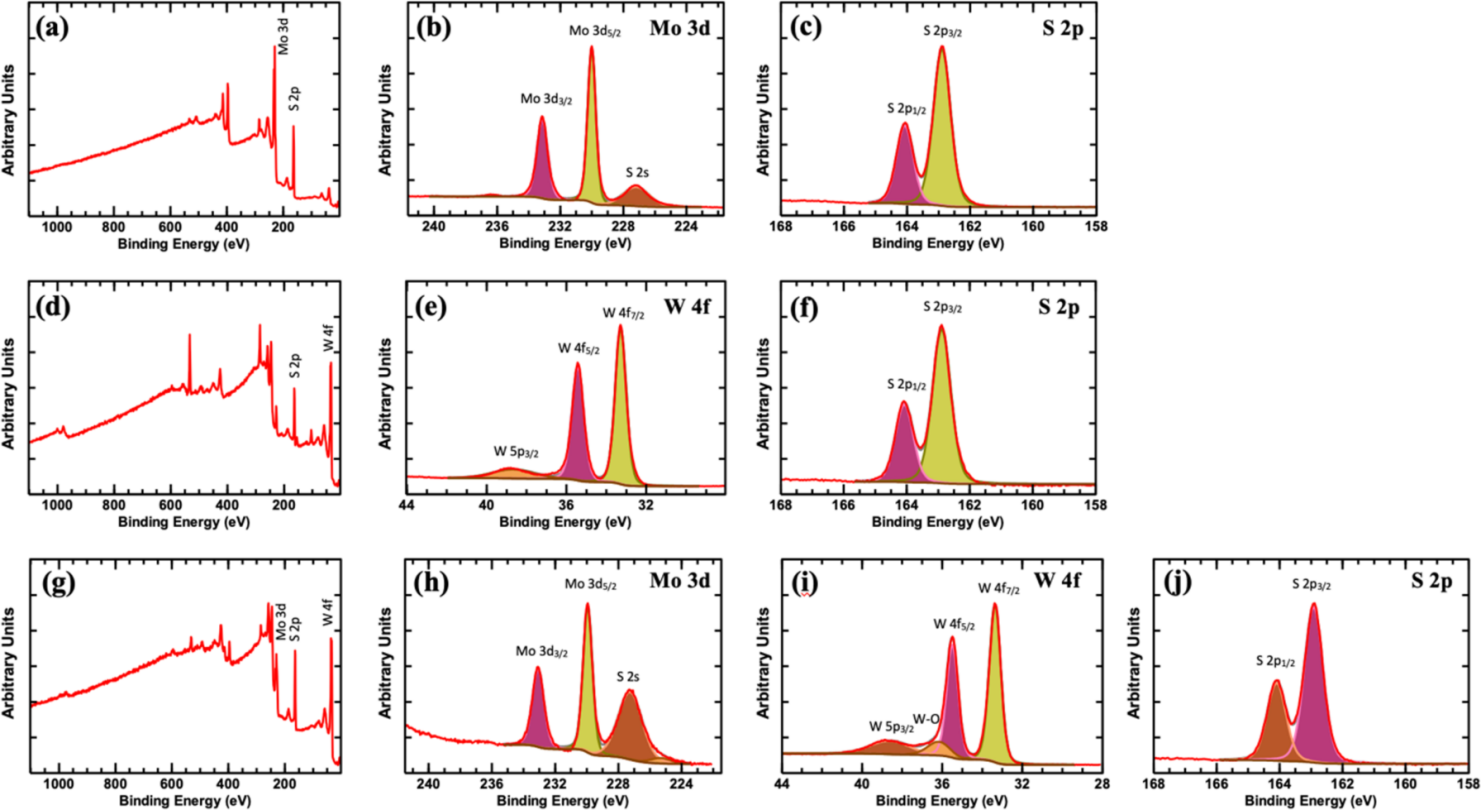
XPS surveys and element edges a–c) MoS_2_, d–f) WS_2_, and g–j) MoS_2_/WS_2_ composite.

The X-ray photoelectron spectroscopy (XPS) survey scans and high-resolution scans for all samples are presented in Figure 3a–j. All XPS analyses were first calibrated using the C 1s peak of carbon at 284.6 eV (Supporting Information File 1, Figure S1). For the MoS_2_ scan, the deconvoluted peaks for Mo 3d show peaks centered around ≈230 eV and ≈233.1 eV corresponding to 3d_5/2_ and 3d_3/2_ peaks of Mo 3d [27–29]. A small peak appearing at around ≈227.2 eV is ascribed to S 2s (Figure 2b) [28]. In addition, the deconvoluted peaks of S 2p appear at ≈162.9 eV and ≈164.1 eV attributed to the S 2p doublet (2p_3/2_ and 2p_1/2_) as shown in Figure 3c [30].

High-resolution scans of W 4f and S 2p are shown in Figure 2e and Figure 2f, W 4f shows deconvoluted peaks at around ≈33.3 eV and ≈35.4 eV corresponding to the W 4f doublet (W 4f7/2 and W 4f5/2) [31]. An additional minor peak appearing at ≈38.7 eV is identified as W 5p3/2 [31]. The XPS analysis of the composite sample indicates the presence of the Mo 3d doublet peaks and S 2s peaks as well as the W 4f doublets, in addition to a peak appearing at ≈36.2 eV ascribed to W–O bonding [31]. This suggests the presence of minor oxidation of the flakes. Further quantitative analysis of XPS data indicates the following atomic compositions: Mo: 34.68%, S: 65.32%, W: 33.98%, S: 66.02%, and Mo: 14.06%, W: 23.70, S: 62.24%, respectively for MoS2, WS2, and MoS2/WS2 composite samples (e.g., Supporting Information File 1, Figure S2). The composition might vary from sample to sample to some extent depending on the efficiency of the sulfurization process. The XPS clubbed spectra for Mo 3d, S 2p, and W 4f indicate that there is no noticeable shifting in the peaks (the binding energies). This means there is no chemical shift in the compounds, implying that the intermixing of Mo and W did not disturb the chemical environment, and the elements retained a stable chemical bond.

### Microstructure analysis

**Figure 4:**
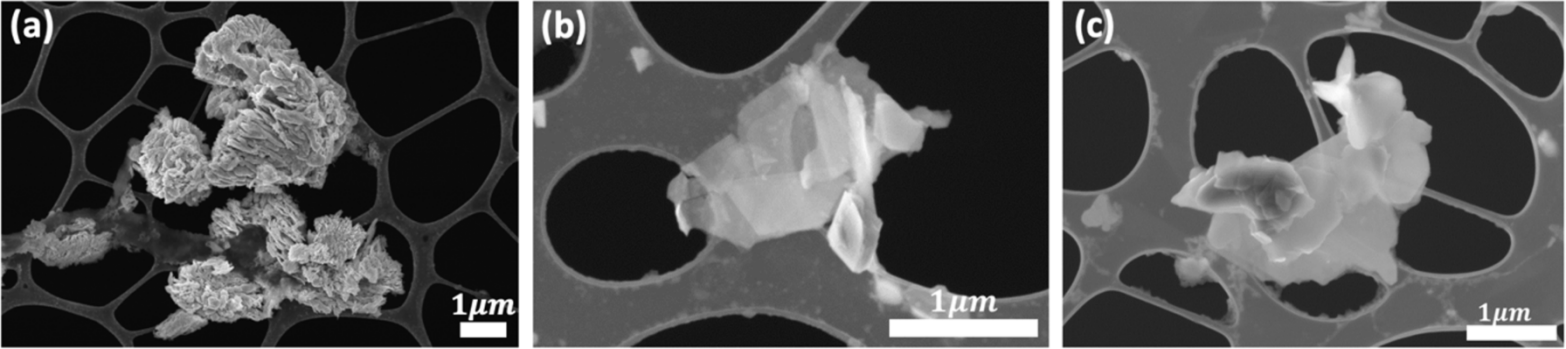
SEM images of exfoliated samples of a) MoS_2_, b) WS_2_, and c) MoS_2_/WS_2_ composite.

Figure 4 shows scanning electron microscopy (SEM) images of all considered samples. The specimens were prepared by drop casting a solution of the exfoliated samples on lacey carbon transmission electron microscopy (TEM) grids. Additionally, agglomeration of the flakes can be usually observed as well, which could be due to the effect of the solvent used for drop casting. The observed flakes have typical shapes such as triangular, hexagonal, pentagonal, and other irregular polygonal shapes. The size of the flakes is within the range of 1 µm. The morphology and the shape of the flakes have common microstructures of MoS_2_/WS_2_ materials grown using chemical vapor deposition (CVD) as previously reported [32]. This means that the samples are well preserved after exfoliation through the intense sonication process. Certain flakes can be observed to have bent. The edges in some flakes can be seen to lose the smoothness, which could be a result of the harsh sonication process. However, the distinct features of the flakes were overall conserved.

**Figure 5:**
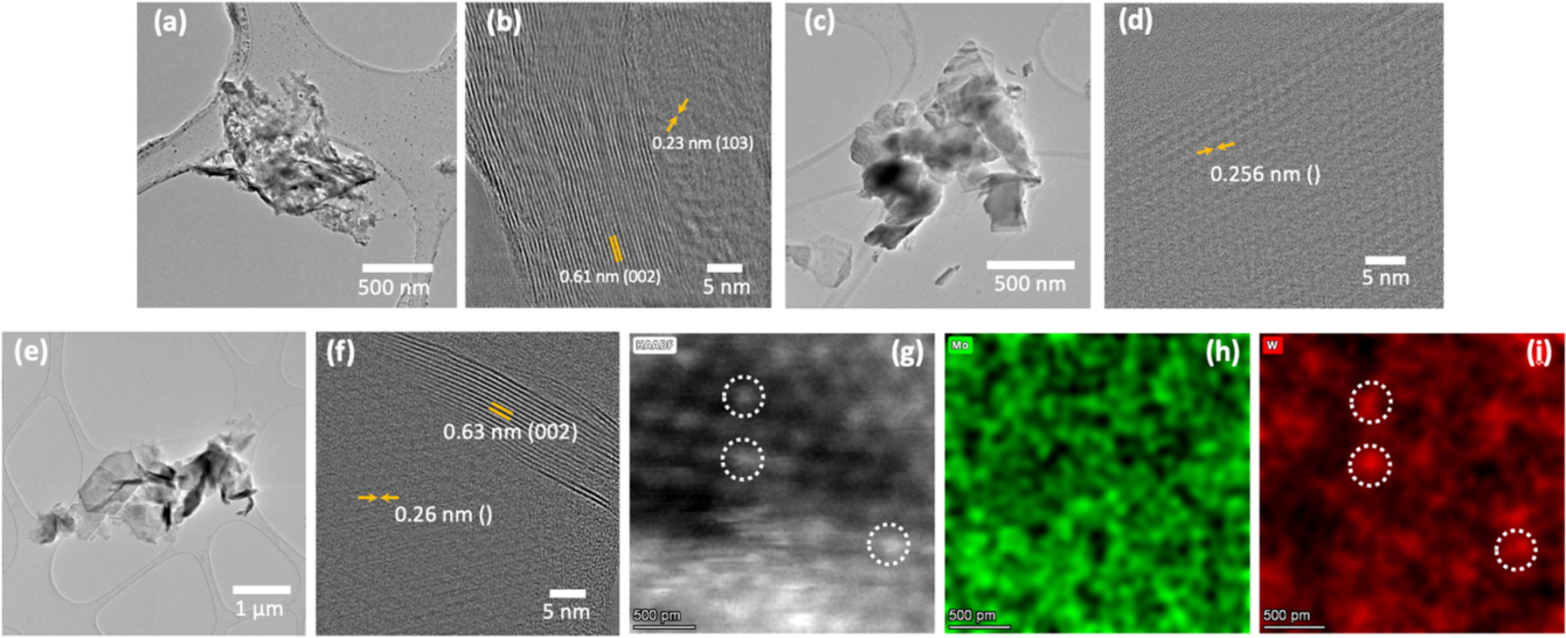
Low- and high-magnification TEM micrographs of a,b) MoS_2_; c,d) WS_2_; and e,f) MoS_2_/WS_2_ composite. g–i) High-resolution STEM-EDS mapping of the MoS_2_/WS_2_ composite depicting a random presence of W atoms (marked by circles).

Figure 5 depicts TEM images carried out on the samples. Low- and high-resolution images captured from MoS_2_, WS_2_, and MoS_2_/WS_2_ composite samples are shown in Figure 5a–f. The low-magnification TEM image indicates that the size of the flakes is in a range of a few hundred nanometers to a few microns, as observed in the SEM images. Figure 5a and Figure 5b show that the MoS_2_ flake has a hexagonal crystal structure with an interplanar distance of 0.61 nm, corresponding to the (002) plane of 2H-MoS_2_ [33]. Other MoS_2_ crystal orientation is indicated by the (103) direction. In the high-resolution image, some edge-related defects can also be seen which is common in CVD-grown MoS_2_ materials [34]. Similarly, the WS_2_ sample exhibits a flake shape along with a high crystalline nature of the flakes (Figure 5c, Figure 5d). Figure 5e and Figure 5f represent the MoS_2_/WS_2_ composite sample. Typical shapes and sizes corresponding to grown MoS_2_ and WS_2_ structures are also observed. High-resolution images indicate different planes corresponding to the composite sample with a corresponding *d*-spacing.

Further, scanning TEM (STEM) images along with energy-dispersive spectroscopy (EDS) mapping were carried out on the MoS_2_/WS_2_ composite. High-annular angle dark-field (HAADF-STEM) allowed the identification of atomic positions with Z differences [35–36], and in particular here, the W sites as shown in Figure 5g. This is confirmed by EDS maps of Mo and W, in Figure 5h and Figure 5i. As it can be seen, the WS_2_ catalyst appeared to be well embedded within the MoS_2_ matrix indicating a successful intermixing of both phases.

### Photodegradation measurements

To examine the photocatalytic performance of MoS_2_ and WS_2_ towards the PD of MB, we first evaluated the PD of the MB dye solution under light excitation without any photocatalysts (MB photolysis) as well as its PD in the dark in the presence of a photocatalyst.

**Figure 6:**
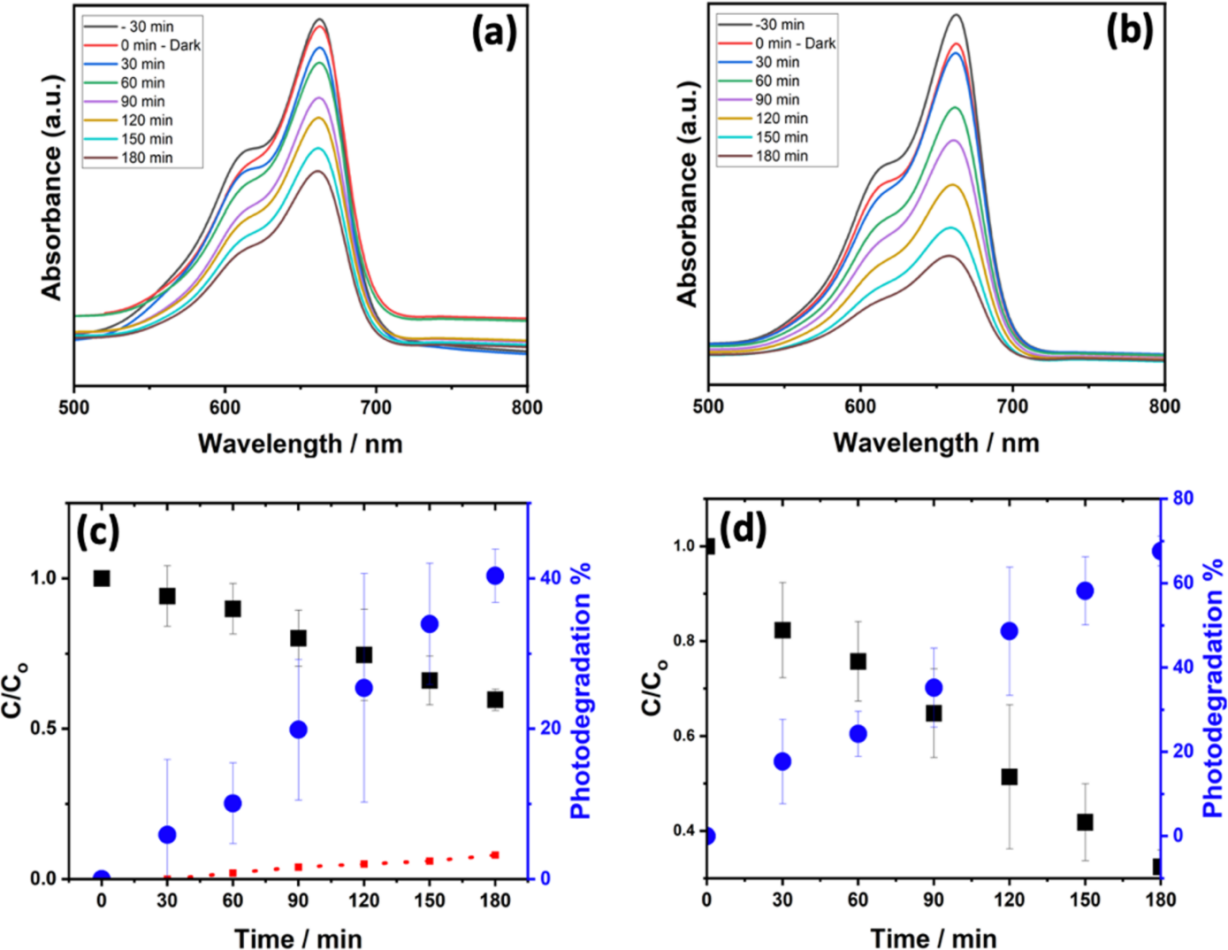
PD of MB recorded under solar simulator excitation: MB absorbance variation at various durations using a) MoS_2_ and b) WS_2_ photocatalysts. The relative MB concentration change during the PD and the corresponding PD efficiency are indicated, respectively in black and blue for c) MoS_2_ and d) WS_2_ photocatalysts. The MB photolysis is amended to c) indicated in red.

The optical absorbance spectra of the MB solution in the presence of MoS_2_ and WS_2_ were recorded in the dark and under visible light illumination at variable exposure durations. The result is displayed in Figure 6a and Figure 6b, respectively. Our findings conform with negligible PD of MB in the absence of light, as observed for both samples 30 min before PD experiments. This minimal MB concentration change recorded in the dark is likely caused by the absorption of MB by WS_2_ [37]. However, upon exposure to light excitation, a considerably greater PD of MB has occurred. As shown in Figure 6c (red dots), there is no direct photolysis of MB, which indicates that the degradation is mainly induced by the presence of MoS_2_ or WS_2_ catalysts. For both catalysts, no isosbestic points are observed in the optical absorption of the solution, suggesting that the MB is completely degraded without the formation of intermediary complexes.

The following equation provides the expression of the PD efficiency at a certain light excitation duration *t* with respect to the MB concentration variation with time.




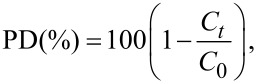




where *C*_0_ (mg/L) is the MB initial concentration in solution, and *C_t_* (mg/L) is the time-dependent MB concentration obtained under light excitation. Figure 6c illustrates the changes in the concentration ratio *C*/*C*_0_ and the PD efficiency under visible light excitation during a 180 min period for MoS_2_. After 180 min of the PD experiment, the initial MB concentration was observed to decrease by 43.5% and 67.6%. in the presence of MoS_2_ and WS_2_, respectively. Further decolorization rate of MB during the photocatalysis was subsequently analyzed using the Langmuir–Hinshelwood kinetics model, expressed by the following equation:




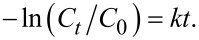




**Figure 7:**
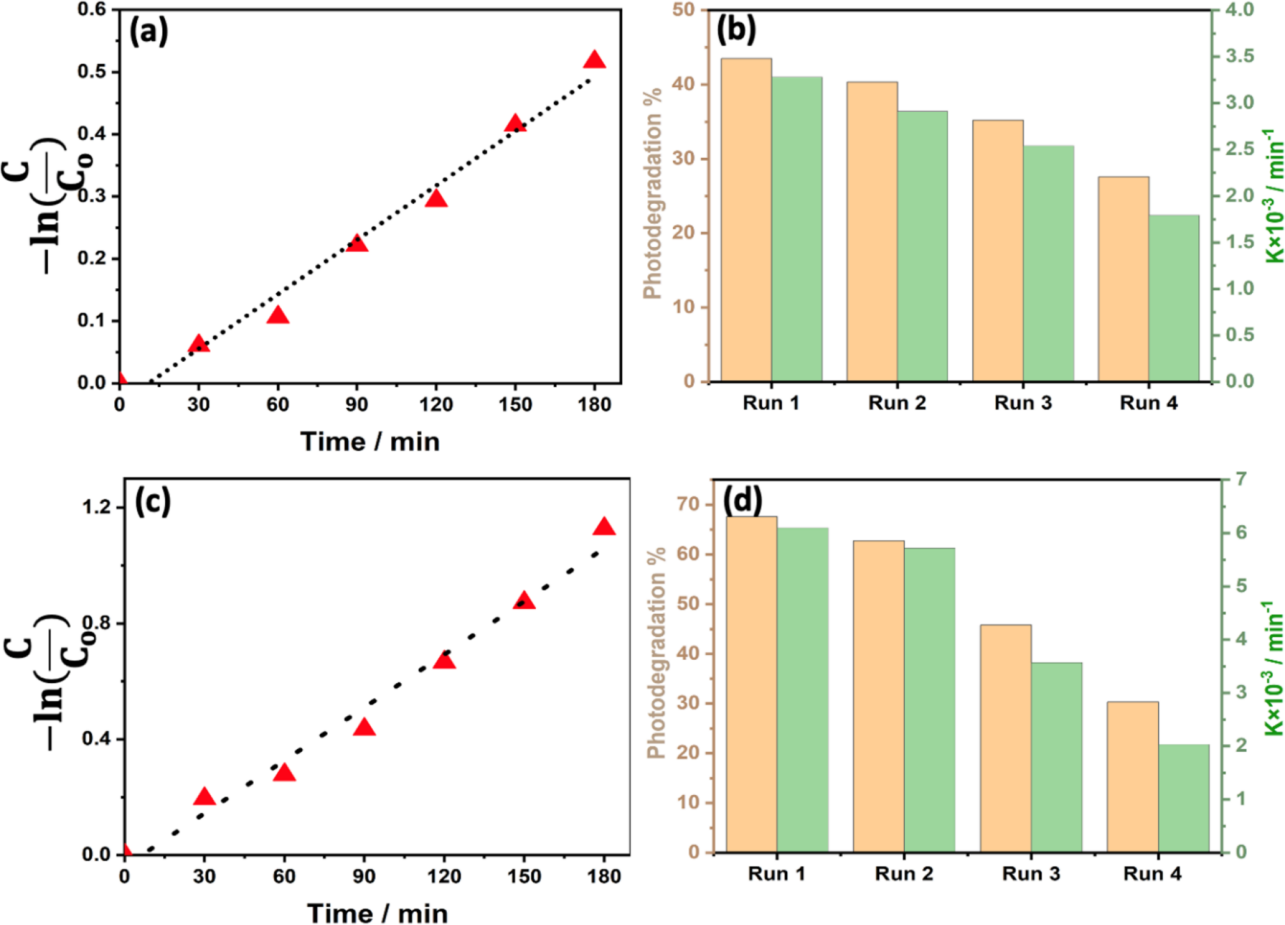
Change in concentration ratio during PD experiments for a) MoS_2_ and c) WS_2_ photocatalysts and the corresponding variation of the PD rate constant (green) and PD efficiency (orange) for four cycles in the presence of b) MoS_2_ and d) WS_2_ photocatalysts.

The PD reaction rate constant, *k*, is the slope of Figure 7a. By plotting −ln(*C*_t_/*C*_o_) as a function of *t*, it was observed that the oxidation of MB using the photocatalyst was well-fitted with the pseudo first-order reaction kinetics model [38]. Our results show a PD rate constant of 6.1 × 10^−3^ min^−1^ and 3.3 × 10^−3^ min^−1^ achieved by WS_2_ and MoS_2,_ respectively.

To evaluate the cyclability of our photocatalysts, we have conducted four consecutive PD runs. After each cycle (3 h), the MB dye solution was replaced with a fresh one to maintain the same initial dye concentration. This means that the remaining dye mass after each PD run was taken into account during the analysis of the results shown in Figure 7b and Figure 7d for MoS_2_ and WS_2_, respectively. To maintain the same concentration of MB, the remaining solution from run one is evaporated. By keeping the same beaker, the residual powder (WS_2_ or/and MoS_2_ + MB) is then diluted using a mixture of fresh MB solution and distilled water to obtain a solution of the same volume and concentration as the run one solution. From one run to the next one, the quantities of MB solution and distilled water required are variable and adjusted experimentally. The redilution process is controlled by the spectrophotometric absorption measurement.

Between the first and fourth cycles, the PD rate constants were observed to decrease from 3.3 min^−1^ to 1.8 × 10^−3^ min^−1^ and 6.1 min^−1^ to 2 × 10^−3^ min^−1^ in the presence of MoS_2_ and WS_2_ photocatalysts, respectively. Similarly, the PD efficiency decreased from 43.5% to 27.6% and 67.6% to 30.3% for MoS_2_ and WS_2_, respectively. These results suggest that despite the very good performance of WS_2_ at the first runs, MoS_2_ has shown a longer lifetime and stability compared to that of WS_2_ [39–40].

**Figure 8:**
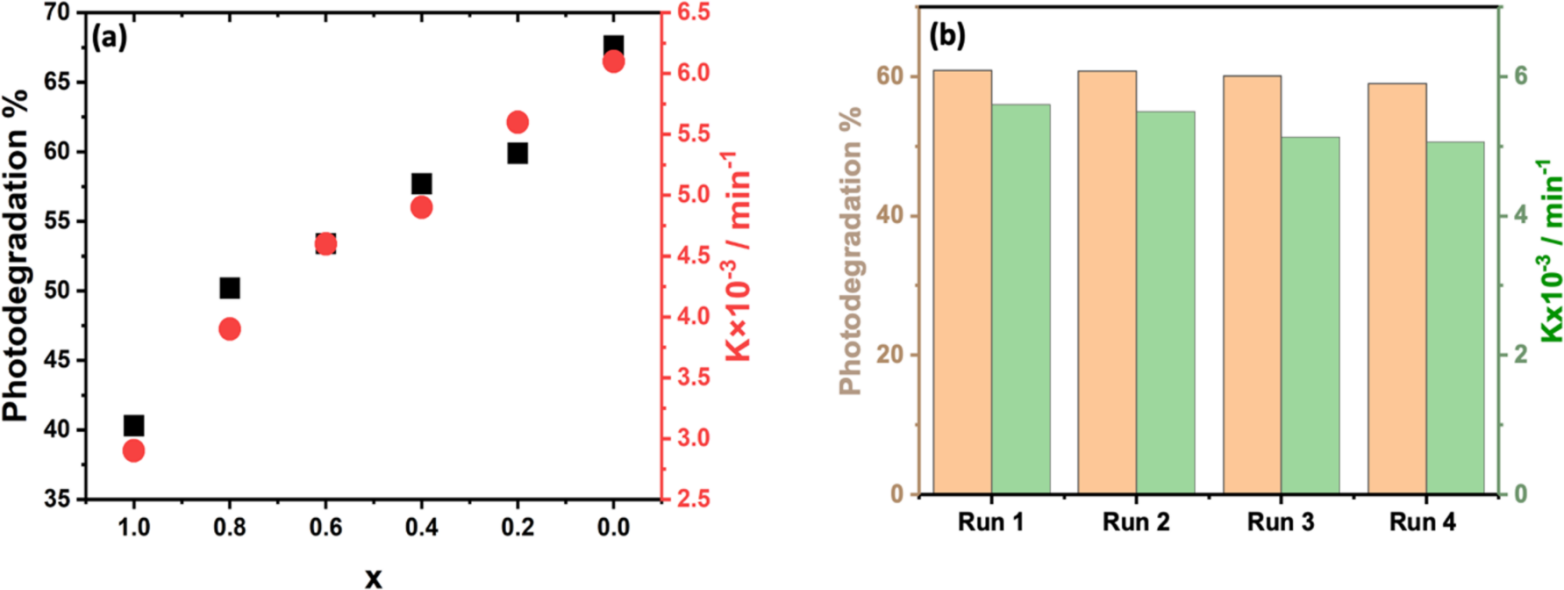
a) PD efficiencies and PD rate-constant variations obtained under solar simulator excitation for (MoS_2_)*_x_*/(WS_2_)_1−_*_x_* photocatalysts at different ratios *x*. b) Selected (MoS_2_)_0.2_/(WS_2_)_0.8_ photocatalyst PD efficiency (orange) and its corresponding PD rate constant (green) for four cycles, the duration of each cycle is 3 h.

To examine the influence of MoS_2_ and WS_2_ intermixing on the PD performance, we have used MoS_2_/WS_2_ intermixing at the following ratios 20%, 40%, 60%, and 80% of WS_2_ toward the PD of MB with the same total amount of catalyst (i.e., 1 mg). Using the PD plots provided in Supporting Information File 1, Figures S3–S5, we extracted and plotted the PD efficiencies and the PD rate constants for all samples in Figure 8a.

As can be seen in Figure 8a, the association of WS_2_ with MoS_2_ has a beneficial impact on the PD performance of MB of the MoS_2_ photocatalyst. The best performance was obtained for (MoS_2_)_0.2_/(WS_2_)_0.8_ exhibiting a PD efficiency of 60% and a PD rate constant of 5.6 × 10^−3^ min^−1^. However, the performances of MoS_2_/WS_2_ mixtures remain inferior to that of pure WS_2_. It can be noted that the blend practically follows the upper limit of the law of mixtures:


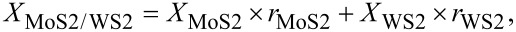


where *X*_MoS2_ and *X*_WS2_ are the same properties for MoS_2_ and WS_2_, and *r*_MoS2_ and *r*_WS2_ the ratios in the mixture. The 100% MoS_2_ showed the lowest PD performance, indicating that the photocatalytic effect of MoS_2_/WS_2_ composites is directed by the presence of WS_2_.

**Figure 9:**
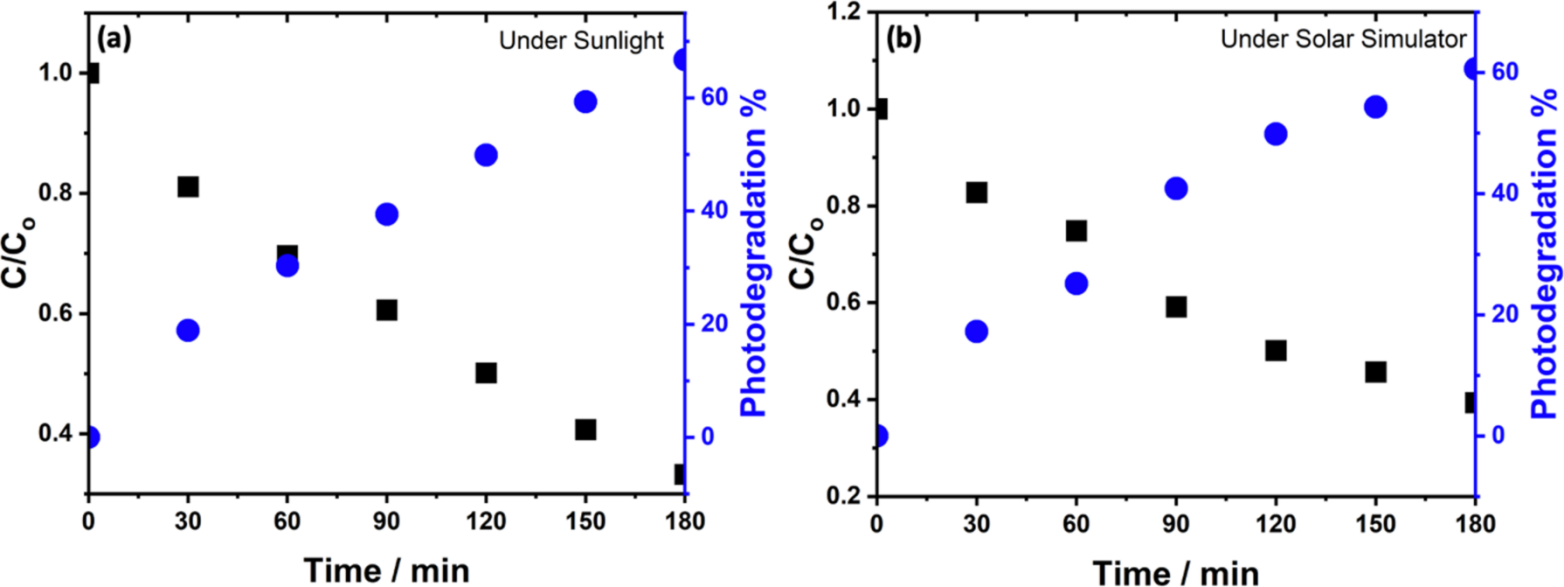
PD efficiency of MB recorded for (MoS_2_)_0.2_/(WS_2_)_0.8_ (blue dots) and the relative MB concentration change during the PD (black squares) using excitations from a) sunlight and b) solar simulator.

Next, we examined the recyclability of the best-performing photocatalyst MoS_2_/WS_2_ for four consecutive PD runs, each cycle lasting 3 h (Figure 8b). The result indicated an excellent long-term stability of the composite photocatalyst suggesting that the association of both MoS_2_ and WS_2_ at the appropriate content, such as (MoS_2_)_0.2_/(WS_2_)_0.8_, not only enhances the overall PD efficiency but also dramatically improves the stability and recyclability of the photocatalyst ascribed to the degradation kinetics. To mimic the real conditions, the optimized sample (MoS_2_)_0.2_/(WS_2_)_0.8_ was selected for PD experiments under direct sunlight (27 °C) in open-sky conditions. Using the same starting concentration of MB and the catalyst quantity, our results showed a higher efficiency of 66.7%, compared to the ones obtained using our solar simulator (Figure 9).

Further decolorization rate of MB during photocatalysis was subsequently analyzed using the Langmuir–Hinshelwood kinetics model as previously explained. Our results show a PD rate constant of 5.97 × 10^−3^ min^−1^ and 5.22 × 10^−3^ min^−1^ achieved under sunlight and solar simulator, respectively, as shown in Supporting Information File 1, Figure S6 and Figure S7).

**Figure 10:**
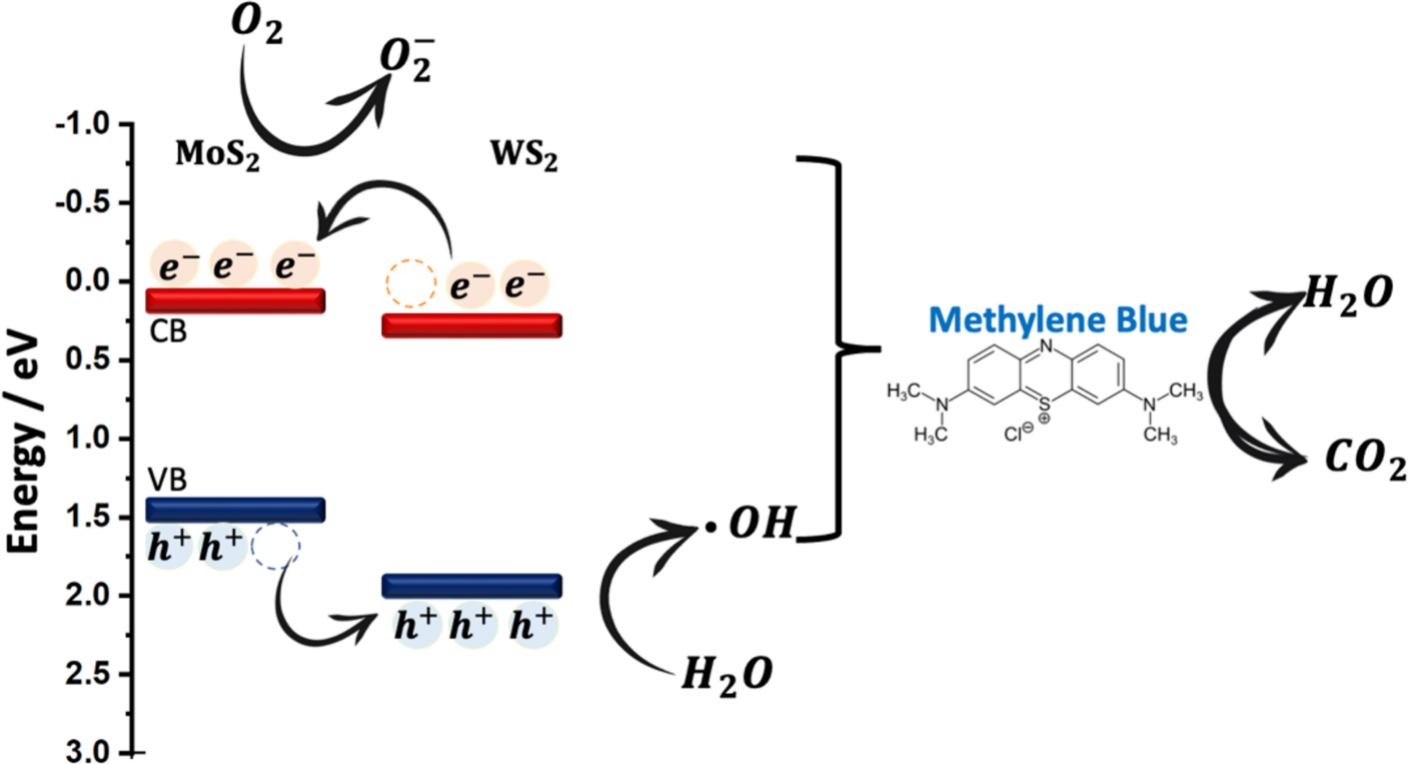
Proposed PD mechanisms of MB by MoS_2_/WS_2_ catalysts.

Generally, the photodegradation of organic pollutants is often driven by reactive agents, such as superoxide radicals, hydroxyl radicals, or photo-induced holes produced from either the conduction or valence bands [41–42]. The mechanism of the PD of MB under visible light excitation consists of several steps: Initially, the MB dye molecules are adsorbed onto the surface of the catalyst [20], then the illumination with energy greater than that of the bandgap will promote electrons (e^−^) to the conduction band (CB), leaving holes (h^+^) in the valence band (VB). Simultaneously, oxygen molecules on the surface of the catalyst capture the excited electrons (e^−^), leading to the formation of superoxide anions (O_2_^−^) [43]. The adsorbed oxygen has the ability to undergo a reaction with two electrons, resulting in the formation of hydrogen peroxide (H_2_O_2_). Hydrogen peroxide subsequently reacts with an electron, forming hydroxyl radicals (•OH), which are required to drive photodegradation reaction. Concomitantly, anion groups serve as electron donors, playing a key role in the reduction process [44]. During the reaction, the VB edge potential of the sample is superior to the standard redox potentials of both •OH/OH^−^ and •OH/H_2_O, suggesting that the holes build up on the VB to generate the oxidization of OH^−^ (see Figure 10), resulting in the formation of •OH. Despite the slow oxidative hydrolysis kinetics, the oxidative radical species remain crucial to drive the photodegradation of organic pollutants [24–25].

Using the Nernst equation [45], the associated energy levels of both CB and VB can be determined. It is worth noting that identifying the *E*_CB_ and *E*_VB_ energy positions is important to comprehend the synergistic effects of MoS_2_ and WS_2_ in the intermixing. Hence, a linear equation is introduced to calculate the energy levels of CB and VB in both materials while taking into account their bandgap energies, as per the following equations:




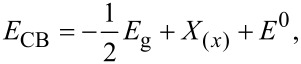







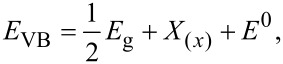




where *E*_CB_ is the energy level of the conduction band, *E*_VB_ is the energy level of the valence band, *E*_g(_*_x_*_)_ and X_(_*_x_*_)_ are the bandgap and the electronegativity of the respective material. *E*^0^ represents the scaling factor that establishes the connection between the absolute vacuum scale and the reference redox level (*E*^0^ = −4.5 eV). The bandgap energies used in our calculation are extracted from our previous work [27]. The obtained results are summarized in Table 1.

**Table 1:** Electronic band structure for both MoS_2_ and WS_2_.

Material	*X*/eV	*E*_g_/eV	*E*_CB_/eV	*E*_VB_/eV

MoS_2_	5.33	1.4	0.13	1.53
WS_2_	5.54	1.57	0.25	1.825

The following schematic diagram depicted in Figure 10 is obtained using the calculated conduction and valence bands positions. The more effective and faster electron transfer kinetics of MoS_2_/WS_2_ should account for the enhanced photocatalytic activity under irradiation.

The PD process can take place as per the following two mechanisms:




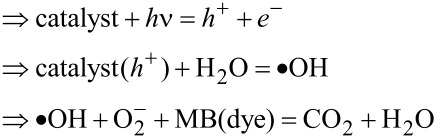




Or as follows:




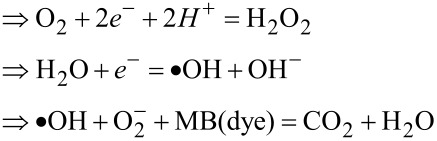




**Figure 11:**
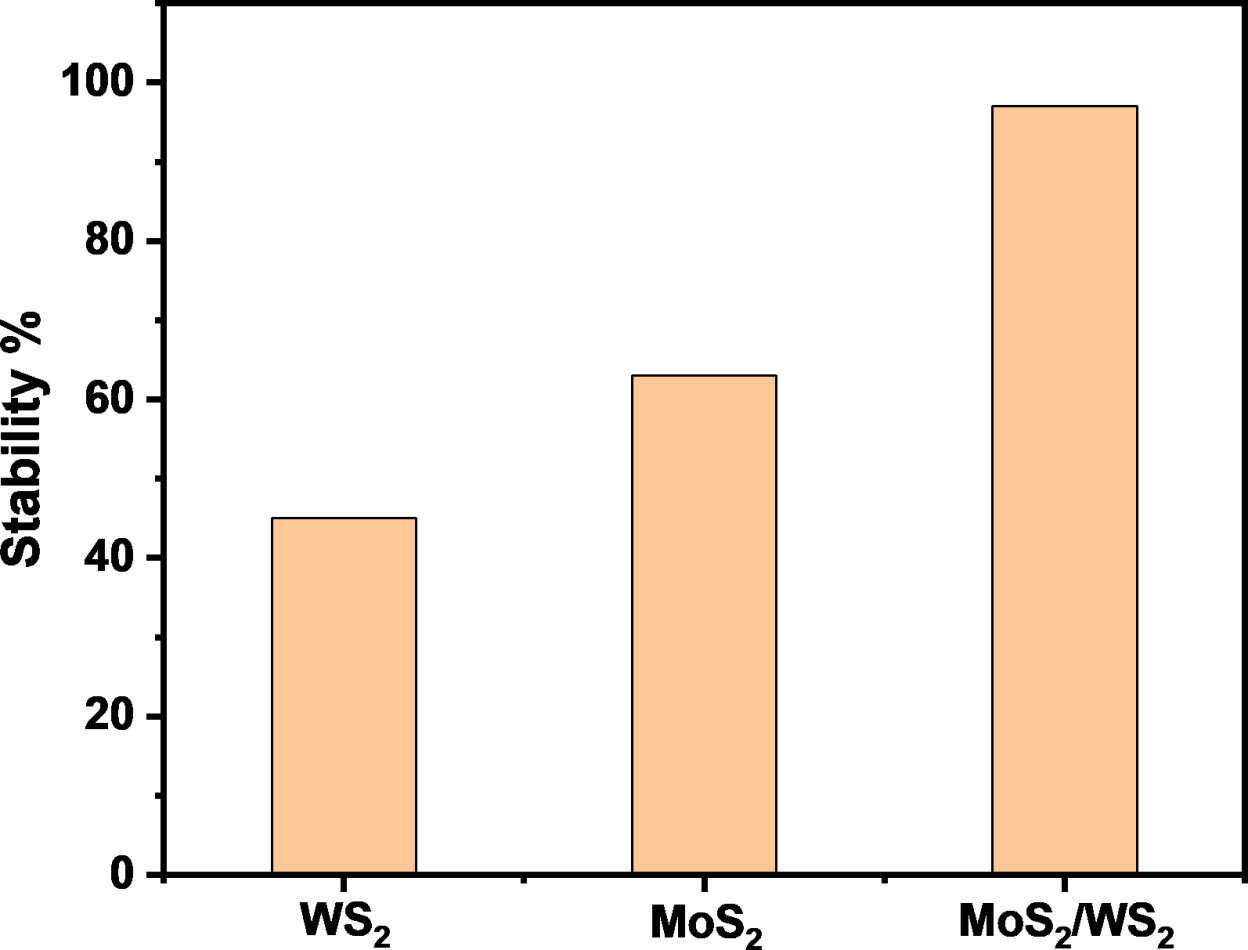
PD stability comparison between WS_2_, MoS_2_ and WS_2_/MoS_2_ composite samples.

The WS_2_/MoS_2_ composite exhibited significant enhancement of PD in terms of long-term stability, reaching up to 97% as shown in Figure 11 compared to that of WS_2_ and MoS_2_ taken alone. We assume this performance is ascribed to the mechanism of PD occurring in the composite sample. Precisely, when the visible light excites electrons in WS_2_ they transition into the CB of MoS_2_ due to band alignment. These photoexcited electrons then move from the CB of WS_2_ to MoS_2_, generating radical electrons that subsequently react with oxygen groups. Simultaneously, photoexcited holes spontaneously migrate from the valence band of MoS_2_ to WS_2_, potentially being captured by water molecules to form hydroxyl radicals. These hydroxyl radicals, in conjunction with valence band holes, contribute to the degradation of MB molecules into CO_2_ and H_2_O, as reported in different studies [42–44]. The effective separation of electron–hole pairs during photodegradation is facilitated by the presence of the MoS_2_/WS_2_ composite. This structure also effectively prevents the recombination of electrons and holes, ensuring an efficient photocatalytic process. In our study, we have employed the facile and cost-effective CVD processing technique to synthesize and intermix MoS_2_ and WS_2_ photocatalysts showcasing a novel approach that distinguishes our work from existing methodologies shown in Table 2, which has shown the best compromise regarding PD performance.

It is worth noting that despite the remarkably low catalyst loading of our CVD-based catalysts (i.e., 1 mg) the obtained PD efficiencies for both MoS_2_ and WS_2_ were relatively high, achieving 43.5% and 67.6%, respectively. This indicates the effectiveness of the CVD method in yielding high-performing photocatalysts. Table 2 compares the present work to previously reported ones. Those authors have used other mixed composites such as MoS_2_-GO and MoS_2_-ZnO prepared by the hydrothermal [22–23] method with photocatalyst loading of 10 to 100 mg, which is ten to hundredfold higher than the ones used for the current PD study.

**Table 2:** Reported MoS_2_ and WS_2_-based composites and their MBs photodegradation efficiency induced by solar excitation compared to this work.

Material	Fabrication	Time (min)	Catalyst (mg)	Concentration (M)	PD %	Ref.

MoS_2_-GO	hydrothermal	60	10	6 × 10^−4^	99%	[22]
MoS_2_-ZnO	hydrothermal	20	15	3 × 10^−5^	97%	[23]
MoS_2_/SnO_2_	hydrothermal	5	20	10^−6^	99.5%	[24]
WS_2_/PPy	polymerization	180	100	3 × 10^−5^	96.2%	[37]
MoS_2_/WS_2_	hydrothermal	180	–	10^−5^	94%	[36]
MoS_2_/WS_2_	hydrothermal	150	100	1.25 × 10^−4^	99%	[46]
MoS_2_/WS_2_	hydrothermal	90	25	1.25 × 10^−4^	85%	[47]
WS_2_@MoS_2_	hydrothermal	120	100	1.25 × 10^−4^	–	[48]
MoS_2_	CVD	180	1	10^−5^	43.5%	this work
WS_2_	CVD	180	1	10^−5^	67.6%	this work
(MoS_2_)_0.2_/(WS_2_)_0.8_	CVD + mixing	180	1	10^−5^	49.6%	this work

Furthermore, this study allowed higher stability of the catalyst when MoS_2_ and WS_2_ were mixed at 0.2 and 0.8 ratios, respectively. Indeed, we noticed that the phase intermixing induced a synergistic effect leading to an enhanced stability of the composite. The low-quantity catalyst loading emphasizes the economic and environmental advantages of our approach, making it a promising avenue for future developments in catalytic materials for pollutant degradation. Overall, these advancements underscore the superior efficacy of the presented synthesis methods, positioning them at the forefront of MoS_2_ and WS_2_ nanocomposite fabrication and paving the way for further advancements in materials science and engineering.

## Conclusion

Neat and intermixed MoS_2_ and WS_2_ phases were evaluated for the PD of MB dye under solar irradiation excitation. The considered samples were systematically characterized by XPS, Raman spectroscopy, SEM, and HRTEM. WS_2_ exhibited the highest PD efficiency and PD rate constant of 67.6% and 6.1 × 10^−3^ min^−1^, respectively. Despite the low PD efficiency achieved, MoS_2_ has shown a very good PD stability of 63%. The intermixed composite (MoS_2_)_0.2_/(WS_2_)_0.8_ showed an enhancement in PD performance and long-term stability by up to 97%. Its evaluation under real conditions under sunlight showed an increased PD efficiency. Overall, our approach is cost-effective, reproducible, and can be further employed in the development of composites processed by CVD, providing a potential solution to address the growing concerns of environmental pollution.

## Experimental

**Figure 12:**
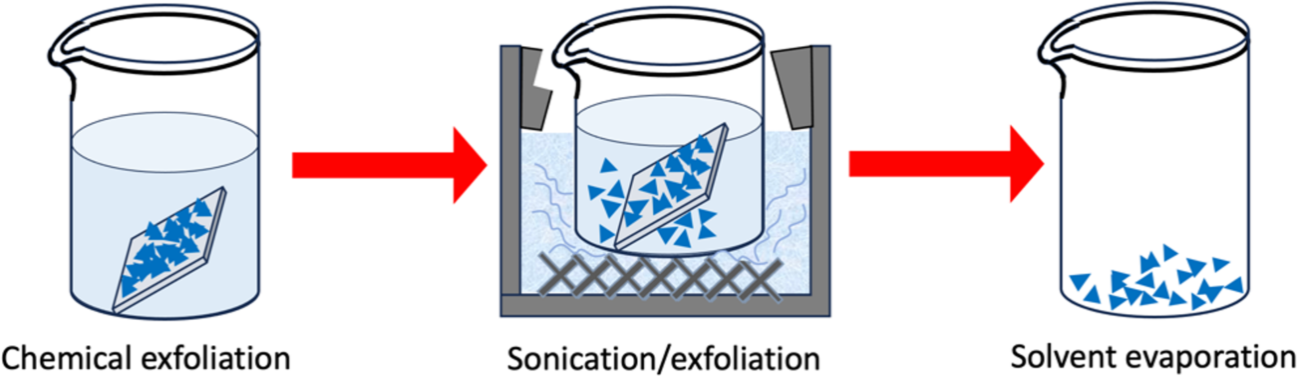
Schematic diagram of the photocatalyst preparation.

Both MoS_2_ and WS_2_ samples were synthesized using a one-step CVD process under atmospheric pressure. The CVD system consists of a quartz tube connected to an argon source. The furnace was gradually heated to specific processing temperatures of MoS_2_ and WS_2_, which were subsequently deposited onto SiO_2_/Si substrates as detailed elsewhere [27]. The fabricated samples were exfoliated in a mixed solution composed of 10 mL of ethanol, 10 mL acetone, and 10 mL of deionized water under sonication for 30 min as shown in Figure 12. Five to ten substrates loaded with the samples were used to prepare the catalyst [49]. The total obtained mass was 200 mg after the exfoliation process and sonication. The obtained material sheets were then extracted from the solution after solvent evaporation, and their mass was determined by subtracting the weight of the sample before and after exfoliation. For all PD experiments, we weighed the samples using a sensitive electronic balance (Secura microbalance, Sartorius) with an accuracy down to 0.01 mg.

Structural, crystalline, and vibrational properties of the fabricated materials were examined utilizing X-ray diffraction (D8 Discover diffractometer, Bruker) with a Kα Cu radiation source at a 1.54 Å wavelength and a micro-Raman spectrometer (Renishaw) equipped with a green laser excitation of 532 nm. The microstructure of the specimen was analyzed using a scanning electron microscope (Thermo Fisher Scientific, Waltham, MA, USA), and a transmission electron microscope (Cs-corrected Titan, Thermo Fisher Scientific). Thin carbon-coated Cu mesh grids were used to prepare TEM samples by the drop-casting method. The surface chemical bonding states and composition of the samples were determined with a scanning XPS microprobe (PHI VersaProbe III, Physical Electronics) equipped with a monochromatic and microfocused Al Kα X-ray source (1486.6 eV). During the experiment, an E-neutralizer (1 V) was implemented. The XPS spectra were calibrated using the C 1s peak position at 284.6 eV as a reference. The CasaXPS software was subsequently used for data processing. The optical properties and photodegradation experiments were conducted on a UV–vis–near IR spectrometer (JASCO V-670) and using solar simulator excitation for the PD monitoring using 10 mL of MB dye solution with a concentration of 5 mg/L (10^−5^ M) and 1 mg of exfoliated photocatalyst. The intensity of the optical absorption peak at 631 nm was used to monitor the concentration of MB in aqueous solution. This peak is commonly associated with photon absorption by the conjugated double bonds of MB molecules. Several measurements conducted on various MB concentrations were analyzed to obtain a correlation between the intensity of the 631 nm peak and the MB concentration. For all experiments, a first run was conducted in the dark for 30 min, followed by PD tests, and maintained for 180 min while monitored by a UV–vis spectrometer every 30 min.

## Supporting Information

File 1 Additional figures.

## Data Availability

The data that supports the findings of this study is available from the corresponding author upon reasonable request.

## References

[1] Chen X, Shuai C, Wu Y, Zhang Y (2021). Environ Impact Assess Rev.

[2] Yang D, Yang Y, Xia J (2021). Geogr Sustainability.

[3] Hao L, Ju P, Zhang Y, Zhai X, Sun C, Duan J, Su Y, Lu Z, Liao D (2021). Colloids Surf, A.

[4] Pandey S, Fosso-Kankeu E, Spiro M J, Waanders F, Kumar N, Ray S S, Kim J, Kang M (2020). Mater Today Chem.

[5] Shahid M K, Kashif A, Fuwad A, Choi Y (2021). Coord Chem Rev.

[6] Soares S F, Fernandes T, Trindade T, Daniel-da-Silva A L (2020). Environ Chem Lett.

[7] Mohammed M A, Shitu A, Ibrahim A (2014). Res J Chem Sci.

[8] Hassanpour M, Safardoust-Hojaghan H, Salavati-Niasari M (2017). J Mol Liq.

[9] Binazadeh M, Rasouli J, Sabbaghi S, Mousavi S M, Hashemi S A, Lai C W (2023). Materials.

[10] Chen C, Ma W, Zhao J (2010). Chem Soc Rev.

[11] Pirhashemi M, Habibi-Yangjeh A, Rahim Pouran S (2018). J Ind Eng Chem (Amsterdam, Neth).

[12] Zong X, Yan H, Wu G, Ma G, Wen F, Wang L, Li C (2008). J Am Chem Soc.

[13] Chang K, Hai X, Ye J (2016). Adv Energy Mater.

[14] Bhandavat R, David L, Singh G (2012). J Phys Chem Lett.

[15] Lu Q, Yu Y, Ma Q, Chen B, Zhang H (2016). Adv Mater (Weinheim, Ger).

[16] Chen S, Takata T, Domen K (2017). Nat Rev Mater.

[17] Liang Z, Shen R, Ng Y H, Fu Y, Ma T, Zhang P, Li Y, Li X (2022). Chem Catal.

[18] Yang R, Fan Y, Zhang Y, Mei L, Zhu R, Qin J, Hu J, Chen Z, Hau Ng Y, Voiry D (2023). Angew Chem, Int Ed.

[19] Ahmaruzzaman M, Gadore V (2021). J Environ Chem Eng.

[20] Joseph A, Aneesh P M (2022). Mater Res Bull.

[21] Amaral L O, Daniel-da-Silva A L (2022). Molecules.

[22] Ding Y, Zhou Y, Nie W, Chen P (2015). Appl Surf Sci.

[23] Ritika, Kaur M, Umar A, Mehta S K, Singh S, Kansal S K, Fouad H, Alothman O Y (2018). Materials.

[24] Szkoda M, Zarach Z, Nadolska M, Trykowski G, Trzciński K (2022). Electrochim Acta.

[25] Kaur N, Singh M, Moumen A, Duina G, Comini E (2020). Materials.

[26] Marmolejo-Tejada J M, Fix J P, Kung P, Borys N J, Mosquera M A (2022). J Phys Chem C.

[27] Turner N H, Single A M (1990). Surf Interface Anal.

[28] Ji H, Hu S, Shi S, Guo B, Hou W, Yang G (2018). J Mater Sci.

[29] Li B, Jiang L, Li X, Ran P, Zuo P, Wang A, Qu L, Zhao Y, Cheng Z, Lu Y (2017). Sci Rep.

[30] Mouloua D, LeBlanc‐Lavoie J, Pichon L, Rajput N S, El Marssi M, Jouiad M, El Khakani M A (2024). Adv Opt Mater.

[31] Mao X, Xu Y, Xue Q, Wang W, Gao D (2013). Nanoscale Res Lett.

[32] Al Qaydi M, Kotbi A, Rajput N S, Bouchalkha A, El Marssi M, Matras G, Kasmi C, Jouiad M (2022). Nanomaterials.

[33] Ochedowski O, Marinov K, Scheuschner N, Poloczek A, Bussmann B K, Maultzsch J, Schleberger M (2014). Beilstein J Nanotechnol.

[34] Rajput N S, Sloyan K, Anjum D H, Chiesa M, Al Ghaferi A (2022). Ultramicroscopy.

[35] Gong K, Lou W, Zhao G, Wu X, Wang X (2020). Friction.

[36] Aswal D, Bamola P, Rani C, Rawat S, Bhatt A, Chhoker S, Sharma M, Dwivedi C, Kumar R, Sharma H (2023). ChemistrySelect.

[37] Shahabuddin S, Mehmood S, Ahmad I, Sridewi N (2022). Nanomaterials.

[38] Jawad A H, Mubarak N S A, Ishak M A M, Ismail K, Nawawi W I (2016). J Taibah Univ Sci.

[39] Rajput N S, Kotbi A, Kaja K, Jouiad M (2022). npj Mater Degrad.

[40] Deokar G, Rajput N S, Li J, Deepak F L, Ou-Yang W, Reckinger N, Bittencourt C, Colomer J-F, Jouiad M (2018). Beilstein J Nanotechnol.

[41] Rajput N S, Shao-Horn Y, Li X-H, Kim S-G, Jouiad M (2017). Phys Chem Chem Phys.

[42] Rajput N S, Baik H, Lu J-Y, Tamalampudi S R, Sankar R, Al Ghaferi A, Chiesa M (2021). J Phys Chem C.

[43] Ji Q, Zhang Y, Gao T, Zhang Y, Ma D, Liu M, Chen Y, Qiao X, Tan P-H, Kan M (2013). Nano Lett.

[44] Wu Y, Gao Z, Sun X, Cai H, Wu X (2021). J Environ Sci.

[45] Sharma R, Uma, Singh S, Verma A, Khanuja M (2016). J Photochem Photobiol, B.

[46] Zhao Y, Liu J, Zhang X, Wang C, Zhao X, Li J, Jin H (2019). J Phys Chem C.

[47] Luo S, Dong S, Lu C, Yu C, Ou Y, Luo L, Sun J, Sun J (2018). J Colloid Interface Sci.

[48] Li H, Yu K, Li C, Guo B, Lei X, Fu H, Zhu Z (2015). J Mater Chem A.

[49] Mouloua D, Lejeune M, Rajput N S, Kaja K, El Marssi M, El Khakani M A, Jouiad M (2023). Ultrason Sonochem.

