# Supplementary material for: Intermixing of MoS2 and WS2 photocatalysts toward methylene blue photodegradation
Source: Beilstein J Nanotechnol. 2024 Jul 5;15:817–29. doi: 10.3762/bjnano.15.68 (PMC11228617; doi:10.3762/bjnano.15.68)
Supplement: File 1 — Additional figures. [file Beilstein_J_Nanotechnol-15-817-s001.pdf]

BEILSTEIN JOURNAL OF NANOTECHNOLOGY

Supporting Information

for

Intermixing of MoS_2 and WS_2 photocatalysts toward methylene blue photodegradation

Maryam Al Qaydi, Nitul S. Rajput, Michael Lejeune, Abdellatif Bouchalkha, Mimoun El Marssi, Steevy Cordette, Chaouki Kasmi and Mustapha Jouiad

Beilstein J. Nanotechnol. **2024,** *15,* 817–829. doi:10.3762/bjnano.15.68

Additional figures

License and Terms: This is a supporting information file under the terms of the Creative Commons Attribution License (https://creativecommons.org/licenses/by/4.0). Please note that the reuse, redistribution and reproduction in particular requires that the author(s) and source are credited and that individual graphics may be subject to special legal provisions.
The license is subject to the *Beilstein Journal of Nanotechnology* terms and conditions: (https://www.beilstein-journals.org/bjnano/terms)

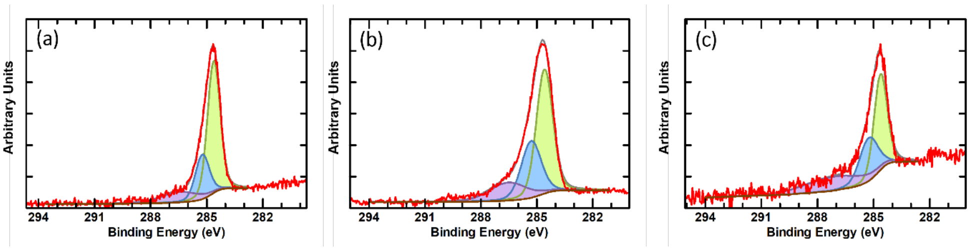

Figure S1: C 1s peaks for XPS calibration for a) MoS_2, b) WS_2, and c) $(MoS_2)_x(WS_2)_{1-x}$ ($0 \leq x \leq 1$)samples.

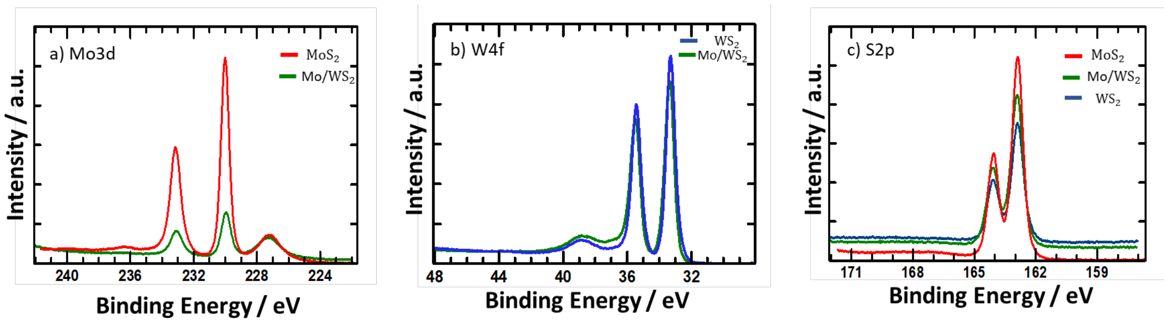

Figure S2: XPS analysis for a) $MoS_{2/Mo3d}$, b) $WS_{2/W4f}$, and c) S2P.

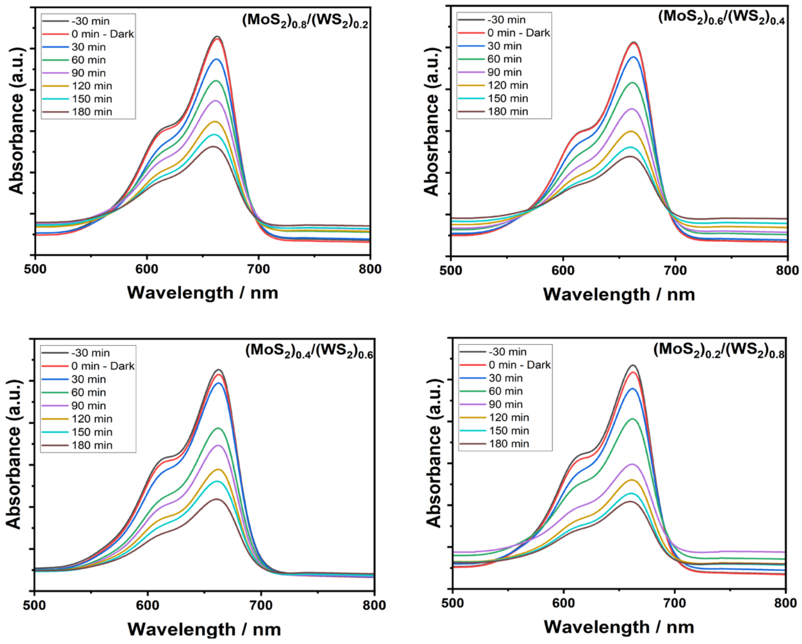

Figure S3: Photocatalysts $(MoS_2)_x/(WS_2)_{1-x}$ ($0 \leq x \leq 1$); MB absorbance variation as a function of time obtained after solar simulator excitation.

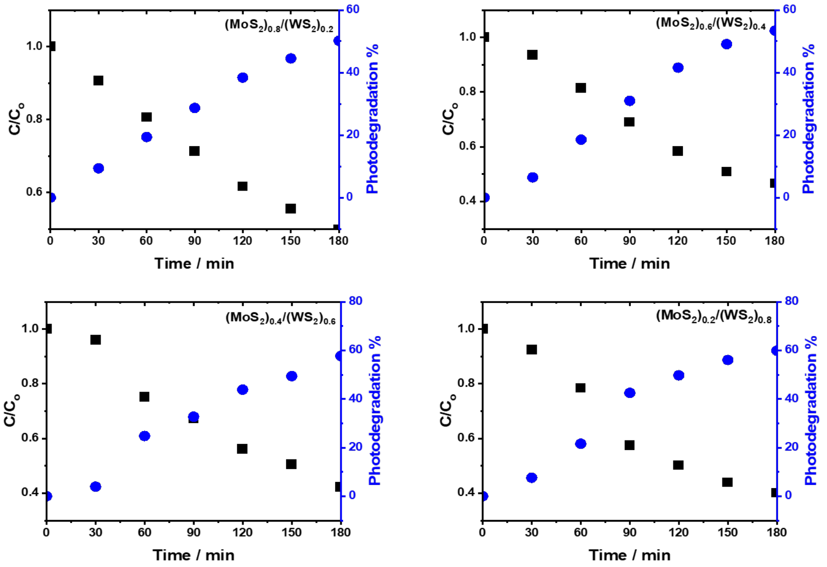

Figure S4: PD of MB by $(MoS_2)_x/(WS_2)_{1-x}$ under solar simulator excitation, relative MB concentration change (black) and corresponding PD efficiency (blue).

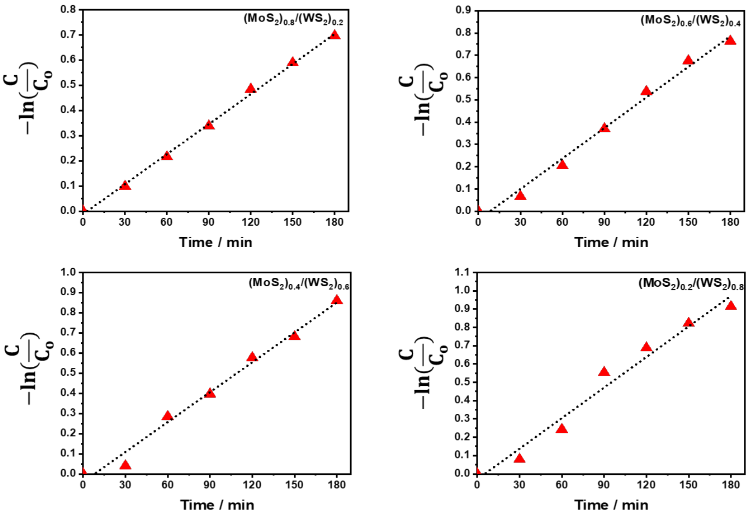

Figure S5: PD rate constant for $(MoS_2)_{0.2}/(WS_2)_{0.8}$. PD experiments carried out under direct sunlight excitation (sunny day in Amiens city, France, 25th of June 2023). Ambient temperature was about 27 °C.

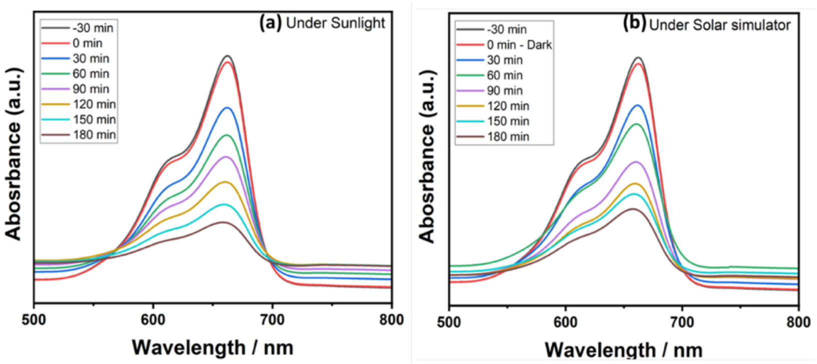

Figure S6: MB absorbance evolution as a function of time for photocatalysts $(MoS_2)_{0.2}/(WS_2)_{0.8}$ after a) sunlight and b) solar simulator excitation.

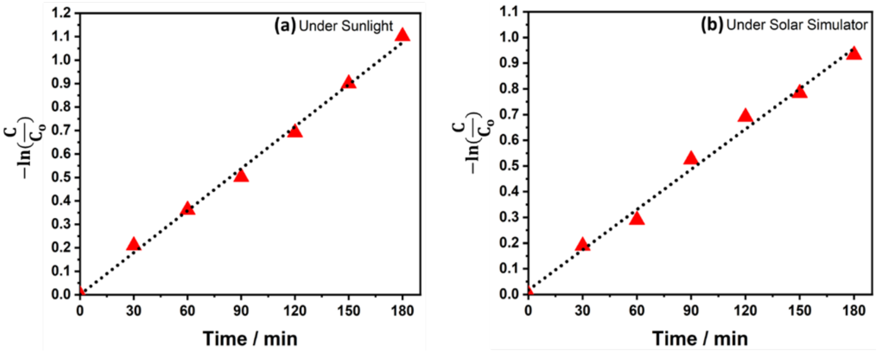

Figure S7: PD rate constant for $(MoS_2)_{0.2}/(WS_2)_{0.8}$ obtained under a) sunlight and b) solar simulator.
